# Early-Life Mild Traumatic Brain Injury Alters Neurodevelopment and Behavior in Mice

**DOI:** 10.1089/neur.2025.0016

**Published:** 2025-06-30

**Authors:** Rachel R. Corrigan, Anna O. Lanier, Emily S. Dresher, Sahibjot Sran, Tracy A. Bedrosian

**Affiliations:** ^1^ Institute for Genomic Medicine, Nationwide Children’s Hospital, Columbus, Ohio, USA.; ^2^ Department of Pediatrics, The Ohio State University College of Medicine, Columbus, Ohio, USA.

**Keywords:** behavior, brain development, critical periods, microglia, mild traumatic brain injury, white matter injury

## Abstract

Approximately 280 children per 100,000 experience closed-head injuries each year, with over 80% being mild in severity. While most children with mild injuries do not require admission to a hospital and recover well over time, some children experience persistent behavioral and cognitive abnormalities that continue into adolescence. Mild traumatic brain injury (mTBI) during early life has potential to disrupt critical developmental processes and lead to long-term consequences; however, the mechanistic underpinnings of mTBI’s effects on brain development remain understudied. Here, we investigated the effects of early-life mTBI on developmental outcomes using a mouse model. Injury was induced on post-natal day 7 by a single weight drop of one of three different impact intensities. Injury resulted in significant white matter loss as measured by myelin basic protein immunoreactivity at 5 days post injury (dpi). There was no change in the extent of Iba1-positive microglial staining at 5 dpi; however, there was increased expression of complement signaling proteins responsible for microglial-regulated synaptic pruning during this time in development. To assess the neurological consequences of mTBI, we examined the development of innate behaviors and ultrasonic vocalization communication. Injured mice were slower to achieve developmental milestones and exhibited altered communication, indicating functional deficits associated with mild injury. Altogether, this study provides evidence for neurodevelopmental consequences of mTBI and demonstrates lasting behavioral effects, suggesting further investigation of mechanisms contributing to neurological effects of mild injury in early life is warranted.

**Figure:**
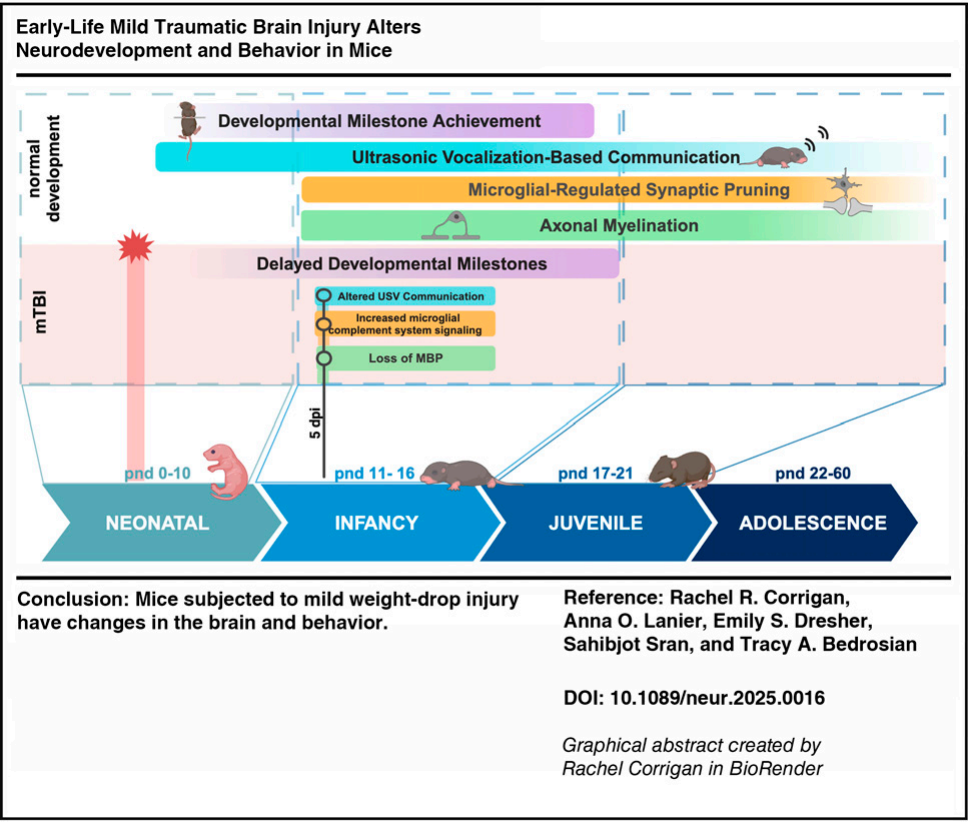


## Introduction

Closed-head injuries affect as many as 280 children per 100,000 each year, with over 80% of injuries considered mild in severity.^1–4^ Between the ages of 0–4 years, the most common causes of traumatic brain injury (TBI)-related emergency department visits are unintentional falls or being struck by or against an object.^5^ While most children with mild injuries have no long-term adverse effects, there is a subset that continues to experience persistent behavioral or cognitive difficulties. A retrospective incidence study evaluating mild TBI (mTBI) occurrences in children 2–17 years of age reported that as many as 14.3% of children who experienced a mTBI still require educational support in the classroom at 12 months post-injury.^2^ Moreover, mild injuries account for a greater share of the overall TBI-related disability burden in 2- to 4-year-old children due to their greater prevalence compared with more severe injuries.^2^ Likewise, adults who experience mTBI have long-term impairments across all cognitive domains.^6^ Unfortunately, not many studies have followed young children who have experienced mTBI longitudinally; thus, specifics about brain dysfunction that could impact a child’s development into adolescence and beyond are largely unknown. A better understanding of mechanisms leading to long-term consequences after mTBI during early life is critical to develop interventions and improve outcomes for children.

Preclinical mouse models of early-life closed-head mTBI are underrepresented in the literature,^7^ possibly due in part to the long-standing view that young brains are more resilient to injury.^8,9^ More recently, it has been appreciated that the developing brain responds differently to injury compared with a mature brain.^10,11^ The first week after birth represents a critical timepoint in murine post-natal neurodevelopment, encompassing rapid changes in cell growth, myelination, and synapse formation.^12,13^ Disruptions or delays, even transiently, in these critical periods of development can disrupt circuit maturation and have been associated with neurodevelopmental disorders.^14–18^ The effects of early-life mTBI on these processes, including behavioral outcomes, are not well understood. Most rodent models of pediatric brain injury have focused on injury at later developmental ages (e.g., post-natal day [pnd] 21), used moderate-to-severe injury paradigms, or used mild impact in the context of repeated concussive events.^19,20^

Thus, we set out to characterize the effects of a pnd 7 mild weight-drop injury in mice from both mechanistic and behavioral perspectives from infancy into juvenile maturity (Graphical Abstract). We hypothesized that mTBI would disrupt critical periods of brain development, causing lasting functional consequences on behaviors developed during early life. To address this, we adapted a previously established pnd 7 single parietal lobe, closed-head, mild weight-drop injury model.^21–23^ Weight-drop injury paradigms in adult rodent models reliably reproduce the cognitive deficits seen after mild injury in humans^24–26^ while closely modeling a closed-head injury. We compared three systematically increasing impact severities to determine the effect on neurological outcomes.

## Methods

### Animals

C57BL/6 mice (Charles River Laboratories) were maintained in a standard 12-h light/12-h dark cycle with access to food and water *ad libitum.* All experiments were performed in compliance with the animal care and use guidelines issued by the National Institutes of Health and approved by the Institutional Animal Care and Use Committee at Nationwide Children’s Hospital under protocol AR20-00080. Experimental mice were bred in-house, and dam and littermates were housed together for the entirety of the experiment.

### TBI paradigm

Male and female mice at pnd 7–8 weighing 3.5–4.0 g were randomly assigned to undergo one of four procedures: sham injury, 0.01 J mTBI, 0.02 J mTBI, or 0.04 J mTBI. The impact intensities were selected based on a range of previously reported mild weight-drop injuries in mice.^27,28^ The injury was induced by weight drop over the parietal cortex as previously described.^21–23^ Briefly, pups were anesthetized with isoflurane, and an incision to expose the skull was made before fixing their head into a stable position in a stereotaxic device. Sham mice underwent this part of the surgery, but no weight was dropped on their skull. Using a Head Trauma Contusion Device for Rat and Mouse, a brass weight was dropped down a hollow cylinder with pinholes onto a 2-mm wide footplate, which was depressed −0.5 mm into the skull, inducing a consistent and reproducible unilateral single hit impact over the right parietal cortex (from lambda: anterior-posterior (AP): 2 mm, medial-lateral (ML): 1 mm, dorsal-ventral (DV): −0.5 mm). Following surgery, the scalp incision was sutured, and pups were placed on a warm heating pad for 10 min before returning to the home cage with their dam.

Approximate kinetic energy (KE, joules) produced from weight-drop impact was calculated under the assumption that in this case, KE equals potential energy (PE). The following equation was used to calculate KE just prior to the impact (PE of the weight and its raised height). *M* represents mass (kilograms), *g* represents acceleration due to gravity (9.8 m/s^2^), and *H* represents height (meters):

KE=MgH

### Tissue preparation

At 5 days post injury (dpi), a cohort of mice was euthanized for tissue collection and randomly assigned for immunohistochemistry (IHC) or Western blotting experiments. For IHC experiments, mice were transcardially perfused with ice-cold phosphate buffered saline (PBS) followed by 4% paraformaldehyde under deep ketamine/xylazine anesthesia. For Western blotting experiments, fresh microdissections of the ipsilateral parietal cortex were collected and immediately snap frozen and stored at −80°C (Supplementary Data S1).

### Immunohistochemistry

IHC was performed on fixed free-floating sections. To characterize the focality of injury in the parietal cortex, —three to four sections per mouse between levels 4.35–4.71 mm posterior from the front of the brain (Atlas of the Developing Mouse Brain)^29^ were examined. Sections were analyzed for white matter (myelin basic protein [MBP]) and reactive gliosis (ionized calcium binding adaptor molecule 1 [IBA1] or glial fibrillary acidic protein [GFAP]) at 5 dpi (Supplementary Table S1, Supplementary Data S1).

Ipsilateral and contralateral sides of the parietal cortex were imaged using brightfield microscopy on a Zeiss AxioImager M2.0 using 5× or 10× objectives. After acquisition, image analysis was performed using ImageJ Fiji (2.14.0/1.54f). Identical ROIs created on ipsilateral and contralateral sides of each histological section were thresholded by selecting Li’s method, one of several automatic thresholding options available in ImageJ,^30^ and the percent area of 3,3'-diaminobenzidine (DAB)-positive immunostaining within the ROI was recorded for each hemisphere. Ipsilateral to contralateral staining ratio was calculated.

### Western blotting

Western blotting of ipsilateral cortices was performed as previously described.^31^ Immunoblots were probed against CX3C chemokine receptor 1 (CX3CR1) and complement system proteins (C3 and C1q; Supplementary Table S1, Supplementary Data S1). Optical density (OD) of each protein was quantified using ImageJ.

### Behavior

All behavioral testing was performed during the light phase by investigators blinded to the experimental condition.

#### Developmental Milestones

Developmental milestones measuring innate behaviors were assessed daily from 1 to 14 dpi (pnd 21–22) using the Hill et al. paradigm^32^ to assess (1) weight and eye opening, (2) innate sensory reflexes, (3) coordination and strength, and (4) locomotion (Supplementary Data S1).

The order and time of day of the assessments stayed consistent throughout testing. Pups were required to successfully complete each test for 2 consecutive days to be considered a “pass,” although post-natal age of day 1 pass was recorded for analysis, with day 2 confirming a learned milestone. After passing a milestone, the behavior was no longer tested in subsequent days for that individual mouse. Behavioral milestone data were analyzed by determining the post-natal day of 100% successful completion for the sham group (per test) and then comparing the percentage of mTBI pups that had also passed on that given post-natal day, creating pass/fail contingency data.

#### Ultrasonic Vocalization Recordings

At 5 dpi, a subset of mice underwent ultrasonic vocalization (USV) recording. Pups in their home cage were habituated to the testing space for 30 min prior to recordings. All recordings were performed in the morning to control for potential time of day differences. Isolation-induced USVs were recorded for 6 min to examine communication behavior post-injury using Avisoft Bioacoustics USV recording equipment and software (Avisoft-RECORDER, version 4.2) by removing the pup from their dam and placing each mouse individually in a sound-attenuating chamber (Med Associates Inc., ENV-022MD-27). Chambers were cleaned with 70% ethanol between each mouse (Supplementary Data S1).

The count of each call typology was recorded for each mouse, and then the percentage of each call typology was averaged per group. Due to inherent variability in the patterns of USV communication, which was observed in both sham and injured mice, we took an additional qualitative approach to gauge patterns of call typologies by grouping simple (one-syllable) type calls and multipart (multisyllable) calls.^33^

### Statistical analysis

Statistical analyses were performed using GraphPad Prism v.10.1.0. Statistical outliers for each data set were determined using Grubb’s test at *p* < 0.05. Body mass was compared among sex and injury groups using a two-way analysis of variance (ANOVA). Male and female mice were pooled for histological and molecular analyses, as no sex differences were observed. All data were checked for normality using a Shapiro–Wilk test and then the Brown–Forsythe test to check for homogeneity of variance. If all groups passed normality, significance among injury groups was evaluated using one-way ANOVA. To examine individual comparisons among injury groups, data were further analyzed using Tukey’s post-hoc test. For data that were not normally distributed, a non-parametric Kruskal–Wallis test with Dunn’s multiple comparisons tests was used. For behavioral milestones testing, pass/fail contingency data tables were analyzed using Fisher’s exact tests for each behavioral test. A *p* < 0.05 was considered statistically significant.

## Results

### Gross response to injury

**FIG. 1.**
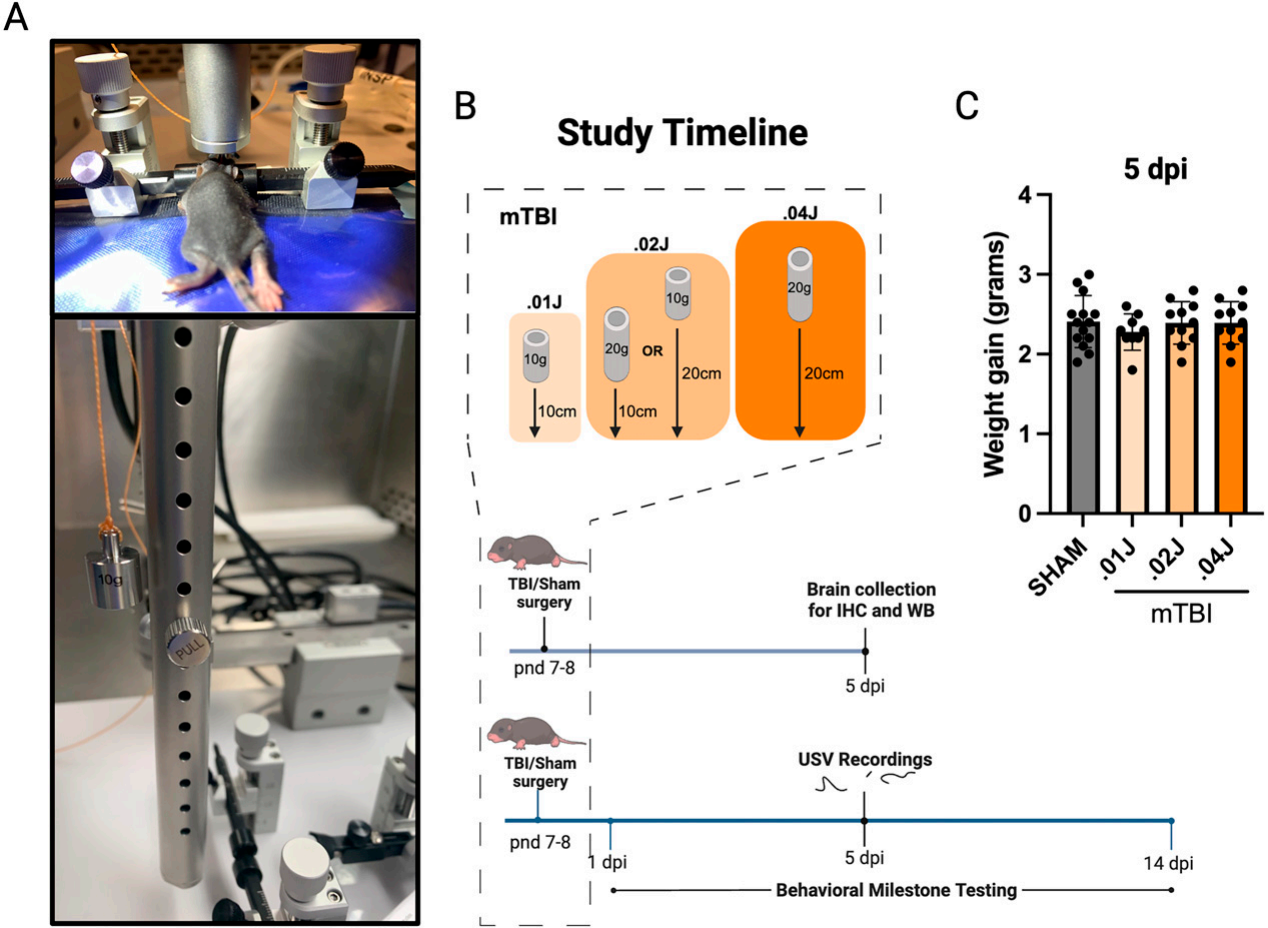
Study design and timelines: **(A)** Post-natal day 7–8 pups were subjected to mild weight drop injuries using the Head Trauma Contusion Device for Rat and Mouse (RWD). **(B)** Mice were randomized to one of four groups including sham (no weight drop, *n* = 63), 0.01 J mTBI (10 g +10 cm drop, *n* = 26), 0.02 J mTBI (10 g + 20 cm drop or 20 g + 10 cm drop, *n* = 78), or 0.04 J mTBI (20 g + 20 cm drop, *n* = 38). Within the 0.02 J mTBI paradigm, both weight drop variables were controlled by equally randomizing mice to receive either double the weight (*n* = 39) or double the height of weight drop (*n* = 39) compared with the 0.01 J mTBI paradigm. **(C)** Weight gain (grams) from day of sham/TBI surgery to 5 dpi. Data are presented as mean ± standard deviation. dpi, days post injury; IHC, immunohistochemistry; J, joules; mTBI, mild traumatic brain injury; pnd, post-natal day; TBI, traumatic brain injury; USV, ultrasonic vocalization; WB, Western blotting.

The weight-drop apparatus, study design, and end-points are depicted in Figure 1A and B. Indicative of a mTBI, there were no signs of skull fracture, apnea, bleeding, cortical lesions, or death from any of the weight-drop injuries. Body mass was measured daily, and there were no differences among sham or injury groups in total weight gained from day of surgery to 5 dpi, *F*(3, 41) = 0.450, *p* = 0.720 (Fig. 1C).

### White matter injury at 5 dpi

**FIG. 2.**
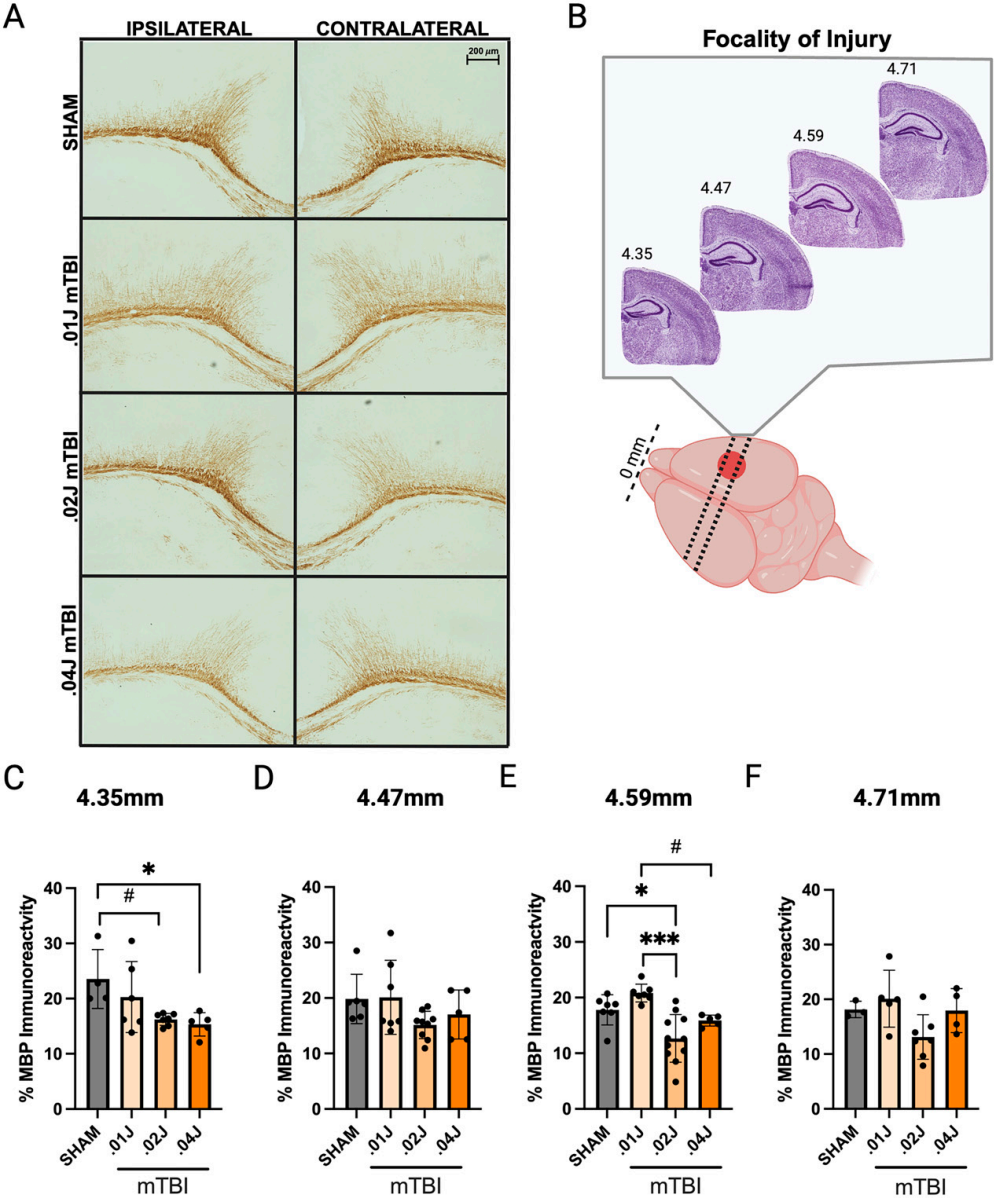
White matter injury at 5 dpi. **(A)** Representative images (10×) of MBP immunoreactivity at anatomical level 4.59 mm showing the corpus callosum and overlying cortex at 5 dpi. **(B)** Schematic showing anatomical levels throughout the injured region individually characterized for MBP staining. **(C)** Percent area of MBP immunoreactivity in the ipsilateral cortex at 4.35 mm (N, sham: 4, 0.01 J mTBI: 6, 0.02 J mTBI: 6, 0.04 J mTBI: 5, outliers: 1), **(D)** at 4.47 mm (N, sham: 6, 0.01 J mTBI: 7, 0.02 J mTBI: 9, 0.04 J mTBI: 5), **(E)** at 4.59 mm (N, sham: 7, 0.01 J mTBI: 7, 0.02 J mTBI: 11, 0.04 J mTBI: 5, outliers: 3), and **(F)** at 4.71 mm (N, sham: 3, 0.01 J mTBI: 5, 0.02 J mTBI: 7, 0.04 J mTBI: 4). One-way ANOVA with Tukey’s multiple comparisons test: #*p* < 0.1, *p < 0.05, **p < 0.01, ***p < 0.001. Data are presented as mean ± standard deviation. ANOVA, analysis of variance; dpi, days post injury; J, joules; mTBI, mild traumatic brain injury; MBI, myelin basic protein.

White matter injury, or damage to myelin sheaths, is commonly observed in closed-head mild injury models.^7^ To gauge the degree of potential MBP loss across increasing mild impact severities, we characterized white matter in the corpus collosum and projection neurons within the ipsilateral and contralateral hemispheres at 5 dpi (Fig. 2A). Three to four sections from each brain sequentially 0.12 mm apart from 4.35 mm to 4.71 mm posterior from the front of the brain were chosen (spanning 0.36 mm) to characterize the anatomical expanse of injury (Fig. 2B).

In the ipsilateral hemisphere, there were significant differences in MBP immunoreactivity across some anatomical levels but not all: 4.35 mm, *F*(3, 18) = 3.912, *p* = 0.0257 (Fig. 2C); 4.47 mm, *F*(3, 23) = 2.002, *p* = 0.142 (Fig. 2D); 4.59 mm, *F*(3, 26) = 10.66, *p* < 0.001 (Fig. 2E); 4.71 mm, *F*(3, 15) = 3.073, *p* = 0.060 (Fig. 2F). At level 4.35 mm, post-hoc comparisons revealed significantly less MBP immunoreactivity in 0.04 J mTBI mice compared with shams (*p* = 0.042) and 0.02 J mTBI mice also showed a tendency toward reduced MBP compared with shams (*p* = 0.055). At level 4.59 mm, post-hoc comparisons revealed reductions of MBP among injured mice: 0.02 J mTBI compared with sham (*p* = 0.010), 0.02 J mTBI compared with 0.01 J mTBI mice (*p* < 0.001), and 0.04 J mTBI compared with 0.01 J mTBI mice (*p* = 0.051). MBP deficits were extended to the contralateral hemisphere (Supplementary Data S2, Supplementary Fig. S1).

### Lack of gliosis at 5 dpi

**FIG. 3.**
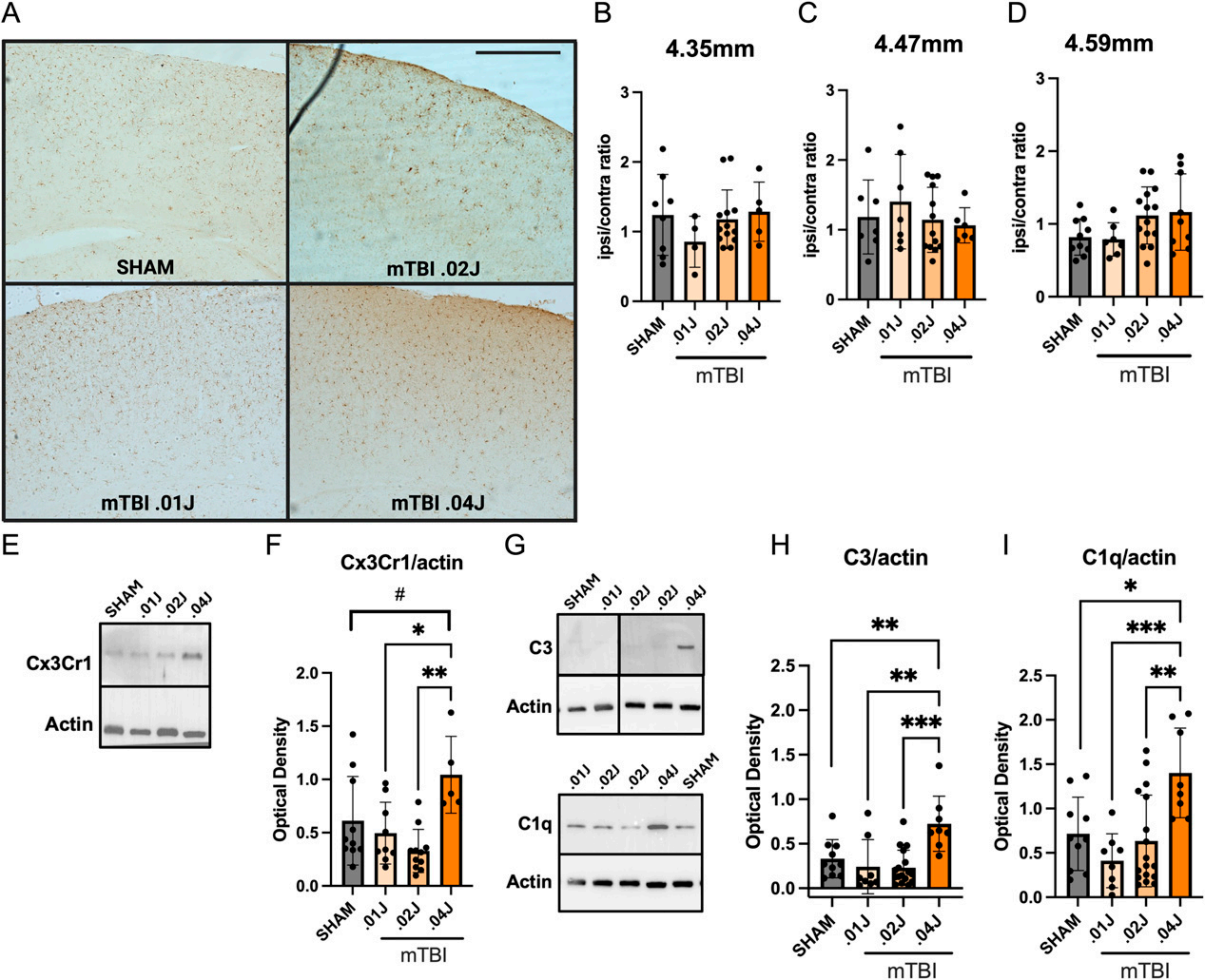
Microglial reactivity at 5 dpi. **(A)** Representative images (10×) of IBA1 immunoreactivity at anatomical level 4.59 mm showing ipsilateral parietal cortex. **(B)** Ipsilateral: Contralateral ratio of percent area IBA1 immunoreactivity at 4.35 mm (N, sham: 8, 0.01 J mTBI: 4, 0.02 J mTBI: 18, 0.04 J mTBI: 5, outliers: 1), **(C)** at 4.47 mm (N, sham: 7, 0.01 J mTBI: 7, 0.02 J mTBI: 13, 0.04 J mTBI: 6, outliers: 1), and **(D)** at 4.59 mm (N, sham: 10, 0.01 J mTBI: 7, 0.02 J mTBI: 14, 0.04 J mTBI: 9, outliers: 1). **(E)** Representative images of cropped immunoblots showing Cx3Cr1 and β-actin visualized at 40 kDa and 43 kDa, respectively. **(F)** Optical density measurements of Cx3Cr1 normalized to β-actin (N, sham: 10, 0.01 J mTBI: 9, 0.02 J mTBI: 11, 0.04 J mTBI: 5). One-way ANOVA with Tukey’s multiple comparisons test: #*p* < 0.1, *p < 0.05, **p < 0.01. **(G)** Representative images of cropped immunoblots showing C3, C1q, and β-actin at 187 kDa, 26 kDa, and 43 kDa, respectively. **(H)** Optical density measurements of C3 normalized to β-actin (N, sham: 9, 0.01 J mTBI: 8, 0.02 J mTBI: 17, 0.04 J mTBI: 8) and **(I)** C1q normalized to β-actin (N, sham: 10, 0.01 J mTBI: 8, 0.02 J mTBI: 17, 0.04 J mTBI: 8). One-way ANOVA with Tukey’s multiple comparisons test: #*p* < 0.1, *p < 0.05, **p < 0.01. Data are presented as mean ± standard deviation. ANOVA, analysis of variance; C3 and C1q, complement system proteins; CX3CR1, CX3C chemokine receptor 1; dpi, days post injury; IBA1, ionized calcium binding adaptor molecule 1; J, joules; mTBI, mild traumatic brain injury.

To characterize gliosis at the site of injury, we evaluated the percent area of IBA1+ microglia and GFAP+ astrocytes within the ipsilateral and contralateral parietal cortex at 5 dpi. We focused this analysis within the region of injury identified through MBP staining: 4.35 mm, 4.47 mm, and 4.59 mm. To determine if subacute microgliosis occurs after mTBI, we stained for IBA1+ microglia in the ipsilateral compared with contralateral parietal cortex (Fig. 3A). Overall, we saw no significant differences in ipsilateral: contralateral ratio of the percent area of IBA1 immunoreactivity at any anatomical level: 4.35 mm, *F*(3, 26) = 0.775, *p* = 0.518; 4.47 mm, *F*(3, 29) = 0.585, *p* = 0.630; and 4.59 mm, *F*(3, 29) = 2.526, *p* = 0.073 (Fig. 3B–D). The IBA1+ percent area quantification for both ipsilateral and contralateral hemispheres is presented in Supplementary Figure S2 (Supplementary Fig. S2A–C). No astrogliosis was observed (Supplementary Data S2, Supplementary Fig. S3).

### mTBI-induced expression of Cx3Cr1, C3, and C1q at 5 dpi

Chemokine Cx3Cl1 signals via the chemokine receptor Cx3Cr1, known to be upregulated after mTBI to elicit an inflammatory response.^34^ We compared Cx3Cr1 expression relative to β-actin among groups using Western blot (Fig. 3E). Cx3Cr1 protein expression was increased, *F*(3, 31) = 5.948, *p* = 0.003 (Fig. 3F), specifically in the 0.04 J impacted mice compared with the milder 0.01 J mTBI (*p* = 0.023) and 0.02 J mTBI (*p* = 0.001) groups.

Around pnd 12–13, microglia and astrocytes have roles in circuit maturation by aiding in synaptic pruning via CX3Cr1-mediated microglial activation but also through regulation of the complement system. The complement system can also be activated after TBI to aid in inflammatory responses.^35–37^ It is unknown whether early-life mild injuries elicit consequences for glial function at critical periods of development, for example, altering the balance of synaptic pruning versus inflammatory cascades.

We examined the overall protein expression of complement proteins C3 and C1q using Western blot (Fig. 3G) and found significant differences among groups within the ipsilateral parietal cortex, *F*(3, 39) = 8.068, *p* < 0.001 and *F*(3, 39) = 7.161, *p* < 0.001, respectively (Fig. 3H–I). Specifically, Tukey’s post-hoc comparisons revealed that 0.04 J mTBI injury mice had significantly higher C3 protein expression compared with 0.01 J mTBI (*p* = 0.002), 0.02 J mTBI (*p* < 0.001), and sham mice (*p* = 0.009). Similarly, 0.04 J mTBI mice had significantly increased C1q protein expression compared with 0.01 J mTBI (*p* < 0.001) and 0.02 J mTBI mice (*p* = 0.002) and sham mice (*p* = 0.020), potentially indicating a 0.04 J minimum threshold of impact severity required to alter expression of complement signaling proteins.

### Delayed developmental milestones

**FIG. 4.**
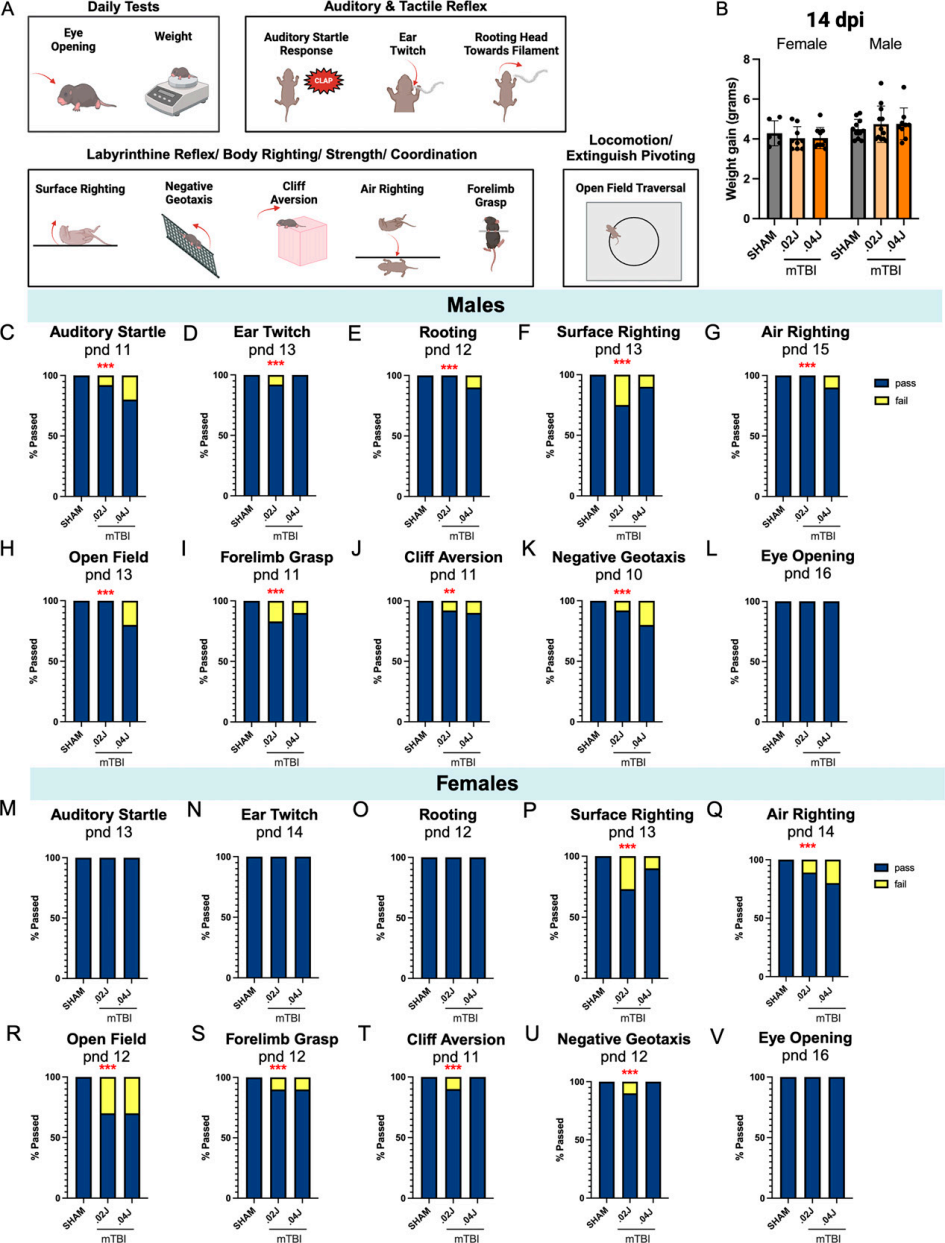
Functional consequences of mTBI: Developmental milestones. **(A)** Schematic showing developmental milestones paradigm tested from 1 to 14 dpi, which included daily tests of auditory and tactile reflexes, labyrinthine reflexes, body righting, strength and coordination, and locomotion. **(B)** Weight gain from date of sham/mTBI surgery to 14 dpi was similar within sexes across groups, although males gained more weight than females overall. **(C–V)** Contingency data are shown for each test comparing pass/fail rates for male mTBI groups compared with sham on the day 100% of sham mice successfully passed. Day of 100% sham pass is shown above each graph. Male 0.02 J mTBI mice were delayed in achieving milestones in 6 of 10 tests compared with sham mice, whereas 0.04 J mTBI males were delayed in 8 of 10 developmental milestones compared with shams. In females, 0.02 J and 0.04 J mTBI mice were delayed in achieving milestones in six and four tests, respectively, compared with sham mice. Notably, female mTBI mice were not delayed in developing innate reflexes (auditory startle, ear twitch, rooting). **(C–L)** Males (N, sham: 11, 0.02 J mTBI: 12, 0.04 J mTBI: 10) and **(M–V)** females (N, sham: 6, 0.02 J mTBI: 10, 0.04 J mTBI: 10). Fisher’s exact test, **p < 0.01, ***p < 0.001. dpi, days post injury; J, joules; mTBI, mild traumatic brain injury; pnd, post-natal day.

We used the Hill et al. behavioral paradigm to evaluate developmental milestones over the first three post-natal weeks (Fig. 4A).^32,38^ As there were no effects of 0.01 J mTBI in prior assays, we proceeded with the two higher injury intensities for the behavioral testing cohort. Daily weight was recorded for each mouse until the study end-point. A two-way ANOVA revealed no weight gain differences between sham and mTBI groups; however, there was a significant difference of average weight gain between males and females justifying evaluation of sex separately for this analysis, injury: *F*(2, 51) = 0.001, *p* = 0.998; sex: *F*(1, 51) = 8.618, *p* = 0.005; interaction: *F*(2, 51) = 0.7609, *p* = 0.473 (Fig. 4B).

To detect developmental delays, we evaluated the percent of injured mice that passed a given test on the post-natal day that 100% of the sham group had passed each task. Fisher’s exact tests revealed that mTBI delayed the achievement of various milestones compared with sham mice. Male mTBI mice exhibited developmental delays in 9 out of 10 tasks: auditory startle (AS): *p* < 0.001 (Fig. 4C); ear twitch (ET): *p* < 0.001 (Fig. 4D); rooting (R): *p* < 0.001 (Fig. 4E); surface righting (SR): *p* < 0.001 (Fig. 4F); air righting (AR): *p* < 0.001 (Fig. 4G); open field traversal (OF): *p* < 0.001 (Fig. 4H); forelimb grasp (FG): *p* < 0.001 (Fig. 4I); cliff aversion (CA): *p* = 0.002 (Fig. 4J); negative geotaxis (NG): *p* < 0.001 (Fig. 4K); eye opening (EO): *p* > 0.999 (Fig. 4L). All injured male mice eventually passed each task, except for AR, for which the 0.04 J mTBI group never reached 100% (*n* = 1 failed).

Female mTBI mice had developmental delays in 7 out of 10 tasks: AS: *p* > 0.999 (Fig. 4M); ET: *p* > 0.999 (Fig. 4N); R: *p* > 0.999 (Fig. 4O); SR: *p* < 0.001 (Fig. 4P); AR: *p* < 0.001 (Fig. 4Q); OF: *p* < 0.001 (Fig. 4R); FG: *p* = 0.001 (Fig. 4S); CA: *p* < 0.001 (Fig. 4T); NG: *p* < 0.001 (Fig. 4U); EO: *p* > 0.999 (Fig. 4V). All female injured mice eventually passed each task, except for SR, where the 0.02 J mTBI group never reached 100% (*n* = 1 failed). For reference, post-natal day of 100% pass for each male and female group is listed in Supplementary Table S2. The magnitude of delay varied by task in both sexes (Supplementary Table S2, Supplementary Data S2).

### Altered USVs

**FIG. 5.**
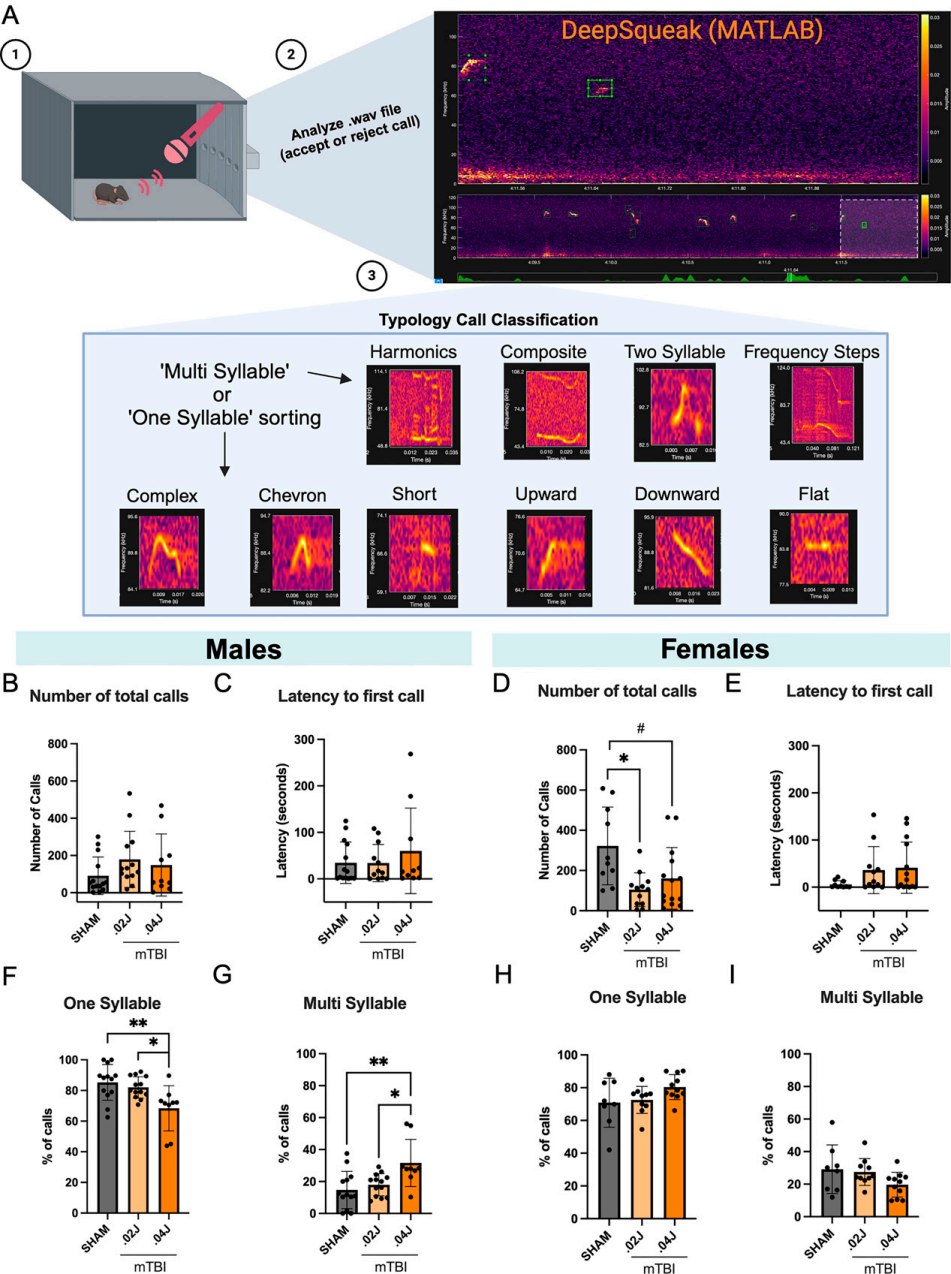
Functional consequences of mTBI: Ultrasonic vocalizations. **(A)** Schematic depicting workflow for USV recordings: (1) Sham or mTBI pups were recorded for isolation-induced USV calls for 6 min, (2) USV waveforms were either accepted or rejected as background noise, and (3) typology call classification was performed, and call types were sorted as either “one-syllable” or “multisyllable.” **(B)** Total number of USV calls produced per group in males (N, sham: 15, 0.02 J mTBI: 13, 0.04 J mTBI: 10, outliers: 2 [1 in number total calls, 1 in latency analysis]). **(C)** Latency to produce the first USV call per group in males. **(D)** Total number of USV calls produced per group in females (N, sham: 10, 0.02 J mTBI: 12, 0.04 J mTBI: 14, outliers: 1 [in latency analysis]). **(E)**. Latency to produce the first USV call per group in females. Kruskal–Wallis test with Dunn’s post-hoc comparisons: *p < 0.05. **(B–E)** Male mTBI mice made similar number of overall calls and displayed similar latency to first call as sham males. However, female mTBI mice made less calls compared with sham females. **(F)** Percentage of “one-syllable” type calls and **(G)** “multisyllable” type calls out of total calls in males. **(H)** Percentage of “one-syllable” type and **(I)** “multisyllable” type calls out of total calls in females. One-way ANOVA with Tukey’s multiple comparisons test: #*p* < 0.1, *p < 0.05. Data are presented as mean ± standard deviation. **(F–I)** Male 0.04 J mTBI mice made less “one-syllable” and more “multisyllable” type calls compared with 0.02 J mTBI and sham male mice, whereas female mTBI mice did not differ in the types of calls produced compared with female sham mice. ANOVA, analysis of variance; dpi, days post injury; J, joules; mTBI, mild traumatic brain injury; pnd, post-natal day; USV, ultrasonic vocalization.

TBI disrupts USV communication in adult mice.^39,40^ Therefore, we investigated whether mTBI early in life influences communication at 5 dpi (Fig. 5A). We recorded isolation-induced USVs during a 6-min trial. Injured male mice had no differences in the number of calls produced or in latency to start producing isolation calls compared with shams (Fig. 5B,C). Injured female mice made less USV calls compared with female sham mice (*H* = 8.722, *p* = 0.013; Fig. 5C). Post-hoc comparisons revealed that female 0.02 J mTBI mice made significantly less calls compared with sham (*p* = 0.013), while 0.04 J mTBI showed a trend toward producing less calls (*p* = 0.073). Latency to first call out after isolation was highly variable among injured female mice (sham: 5.99 ± 7.77 s, 0.02 J mTBI: 36.22 ± 49.93 s, 0.04 J mTBI: 41.2 ± 54.4 s) but not significantly different from sham controls (*H* = 2.631, *p* = 0.270; Fig. 5E).

Recent studies have shown that mice make more multipart syllable calls as they develop into adulthood^41,42^; therefore, we asked whether mTBI would alter the developing mouse lexicon. Whereas injured male mice made the same average number of calls as sham mice, the composition of call types was different from shams (Supplementary Table S3). Injured male mice produced less one-syllable type calls and more multisyllable type calls compared with male shams, *F*(2, 32) = 6.568, *p* = 0.004 (Fig. 5F,G). Post-hoc comparisons revealed that the 0.04 J mTBI group exhibited these differences in call types compared with both sham (*p* = 0.004) and 0.02 J mTBI (*p* = 0.022) mice. Conversely, although injured female mice made less overall USV calls, the composition of call types was similar to sham controls (Supplementary Table S4). Female injured mice produced similar proportions of one- or multisyllable call types compared with uninjured sham controls, *F*(2, 27) = 2.423, *p* = 0.1084 (Fig. 5H,I).

## Discussion

Here, we investigated the consequences of an early-life mild weight-drop injury in mice. Post-natal day 7 in mice is thought to represent full-term infancy in humans, with pnd days 11–19 largely mimicking developmental milestones 0 to <2 years of age^11,43^; however, these comparisons are approximate. We found that mTBI alters the expression of proteins implicated in the developmental functions of glial cells, induces white matter injury, and disrupts behavioral development, with generally worsened outcomes in response to the 0.02–0.04 J impacts compared with 0.01 J impact. These results demonstrate that mild injury in early life can have important effects on the developing brain with functional consequences.

### White matter injury at 5 dpi

We characterized three grades of impact severity by examining white matter loss, which is commonly observed in other models of TBI. In mice, neuronal myelination occurs within a critical period^44,45^ starting at pnd 11 and peaking from pnd 14 to 45,^12^ thus, we set out to determine how mTBI impacts myelination at 5 dpi (pnd 12–13). Our highest injury grades had ∼5–10% reduction of MBP staining, consistent with more severe impact TBI models reporting diminished myelin integrity after experimental TBI at young ages, though we observed mild effects with variability across anatomical levels.^46,47^ A previous model similar to our lowest injury grade found 50–70% loss of myelin at 5 dpi; however, the study used OF1 mice,^22,23,48^ whereas our study examined C57BL6/J mice. Natural variations in skull thickness or curvature could influence the impact force and resulting injury. The differences in background strain could explain the differences in severity of white matter injury. Future studies should compare additional markers of white matter injury in these models.

To further characterize the neuropathology from early-life mTBI at 5 dpi, we assessed gliosis at subacute timepoints but did not see significant signs of reactive glial cells around the injury site. In agreement with our findings, a recent report observed only acute gliosis in a pnd 7 weight-drop model (1–2 dpi) but highlighted a significant microglial inflammatory response as late as 3 weeks post injury.^48^

### Glial mechanisms altered by mTBI

Microglia and the innate immune system have a role in strengthening neuronal circuitry during development.^49^ Besides playing a role in regulating microglial inflammatory responses after a TBI, the complement system plays an important role in synaptic pruning during the second and third weeks of post-natal maturation.^13,50,51^ Increased synaptic density, therefore imbalanced pruning, has been reported in adolescent TBI; however, these studies failed to look at microglial-specific dynamics.^52,53^ Our highest injury grade induced increased expression of the chemokine receptor, Cx3Cr1, compared with sham and lower injury grades at 5 dpi. Additionally, these mice also expressed significantly higher levels of complement proteins C3 and C1q during a developmental period critical to synapse elimination. A limitation of this study is that other cell types in the brain, including astrocytes and endothelial cells, can express these markers, so future studies should investigate cell type-specific expression levels. While our findings here provide a limited snapshot of glial response to injury, they agree with the upregulation of the complement system that has been observed after TBI in human brain samples,^54^ as well as controlled cortical impact rodent models.^36^ Microglial-mediated dysregulation of synaptic pruning during development may have lasting effects on overall circuit maturation and could be a link to behavioral consequences.

### Neonatal mTBI alters behavioral development

Developmental milestones reached from infancy into childhood are critical metrics to screen for developmental delays.^55,56^ We tested a battery of behavioral tasks aimed at gauging the proper development of several innate reflexes as well as strength, coordination, and locomotion.^32^ Early-life mTBI induced developmental delays in both sexes, as well as sex-specific delays in innate sensory reflexes, which were observed in males but not females. In most tasks, there were more mice exhibiting delays in the 0.04 J injury group compared with 0.02 J injury group. In a few tasks, the 0.02 J group demonstrated more delays. However, the magnitude of delay was associated with impact severity. Specifically, in males, 0.04 J mTBI mice were delayed by 3 days in the forelimb grasp, 2 days in the negative geotaxis test, and at least 1 day delayed in most other tasks compared with sham mice, whereas 0.02 J injured mice were never more than 1 day delayed in any developmental task. On average, these differences emerged around 5 dpi (average: pnd 12, range: pnd 11–15), which corresponds to the period in which histological and biochemical changes were observed in the brain.

Communication is a vital marker of normal development. Pups yet to be weaned will elicit isolation-induced calls when separated from their dam, which can serve as a marker for proper communication.^57,58^ At 5 dpi, we found that injured female mice made less USV calls, but the composition of call types was similar to shams. In contrast, injured males made the same number of calls, but composition was different, making more “multisyllable” type calls compared with sham controls. Mouse models of autism spectrum disorder targeting deficiencies of neuroligins and other synaptic cell adhesion molecules make less frequent isolation-induced calls overall but exhibit increased proportions of harmonic, two-syllable, and composite type calls,^33,59,60^ suggesting a link to disrupted brain development.

## Conclusions

This study highlights cellular and behavioral consequences of early-life mTBI. Our findings further support the rationale that injury in early life may disrupt critical periods of brain development and have lasting consequences on behavior. Furthermore, this study offers a comparison of how subtle, yet progressive increases in impact severity while remaining within the bounds of mild injury can have unique outcomes at the behavioral level. While mTBI-induced changes were observed at the molecular level in this study, higher thresholds of injury may be necessary to observe systematic scale differences in outcomes. Regardless, this study shows that while neuropathological findings in the brain were minor at 5 dpi, consequences in behavioral performance can develop from mild injuries early in life. Future studies should investigate cellular and molecular changes over additional developmental timepoints.

## Transparency, Rigor, and Reproducibility

A sample size of eight mice per group was planned based on an expected effect size of 0.80 for the histological and Western blot primary outcomes, calculated to yield 95.6% power to detect significant effect of the one-way ANOVA (primary statistical outcome) with a *p* value <0.05 (G*Power 3.1). A sample size of nine mice per group was planned based on an expected effect size of 0.80 for behavioral primary outcomes, calculated to yield 95.5% power to detect significant effect of the one-way ANOVA (primary statistical outcome) with a *p* value <0.05 (G*Power 3.1). Two hundred and nine mice were subjected to experimental injury, 0 were excluded for technical reasons, 4 died from anesthesia before TBI impact, and 205 were randomly assigned to groups using a blinded external experimenter. Overall, 19 litters were used; pups were equally distributed to each experimental group from each litter. Histological data were performed in three batches, and samples were randomly assigned to each batch. For histological and Western blot data, consistency of between surgical batches was assessed and found to be indistinguishable. The normal distributions of molecular protein and behavioral outcomes were verified with Shapiro–Wilk tests. Statistical outliers were defined using Grubb’s outliers test, set at *p* value <0.05. Behavioral outcomes were all measured starting at 9:30 a.m. (lights on at 6 a.m.). All behavioral outcomes were also analyzed for consistency between litters as well as biases for sound chamber placement in USV analysis.

## Abbreviations Used

CX3CR1: CX3C chemokine receptor 1
GFAP: glial fibrillary acidic protein
IBA1: ionized calcium binding adaptor molecule 1
KE: kinetic energy
MBP: myelin basic protein
mTBI: mild traumatic brain injury
TBI: traumatic brain injury
PE: potential energy
USV: ultrasonic vocalization
