# Supplementary material for: Early-Life Mild Traumatic Brain Injury Alters Neurodevelopment and Behavior in Mice
Source: Neurotrauma Rep. 2025 Jun 30;6(1):465–79. doi: 10.1089/neur.2025.0016 (PMC12270539; doi:10.1089/neur.2025.0016)
Supplement: Supplementary Data [file neur.2025.0016_supplementarydata.docx]

**SUPPLEMENTAL MATERIAL**

**Supplemental Methods**

**Tissue Preparation**

Mice were transcardially perfused with 4% paraformaldehyde for immunohistochemistry (IHC) experiments. Brains were post-fixed in 4% paraformaldehyde overnight, transferred into 30% sucrose solution for cryoprotection, and sectioned coronally at 20 µm on a freezing microtome (Leica, SM2010R). Tissue sections were stored in a 48 well plate in PBS with 0.3% sodium azide at 4°C until immunohistochemical analysis. Fresh frozen microdissections of ipsilateral parietal cortices were used to generate protein homogenates by pestle homogenization of fresh frozen samples in a lysis buffer containing 10X lysis buffer, dithiothreitol (DTT) (1:200), phenylmethylsulphonyl (PMSF) (1:1000), protease inhibitor cocktail (PIC) (1:200), and phosphatase inhibitors (1:50). BCA protein assay was performed to determine the protein concentration of each sample.

**Immunohistochemistry**

Briefly, antigen retrieval was performed by incubating sections in a 1X citrate buffer at 85$^{\circ}$C for 30 minutes (Sigma-Aldrich). Endogenous peroxidase activity and nonspecific antibody binding were blocked with 0.3% hydrogen peroxide for 30 minutes and 4% donkey serum, respectively. Sections were then transferred to primary antibody solution and incubated at 4°C overnight. Sections were washed and incubated in biotin conjugated secondary antibody for 2 hours, followed by ABC Reagent (Vector Labs PK-4000). Visualization of reactivity was achieved by DAB (3,3′-Diaminobenzidine) (Vector Labs, SK-4100). Sections were next mounted on gelatin-coated slides using EZ Float buffer (EMD Millipore, NS350-5ML) and left to dry overnight. The following day, sections were dehydrated through a series of water, 70% ethanol, 90% ethanol, 100% ethanol, and xylenes washes and then slides were mounted with VectaMount Express Mounting Medium and cover slipped.

**Western Blotting**

Briefly, 15 µg protein/sample were run through SDS-PAGE on a 10-20% polyacrylamide gel at 120V for 90 minutes and transferred onto 0.2 µm PVDF membranes using a Trans-Blot Turbotransfer System (Biorad). Blots were then blocked against non-specific binding with 10% milk in TBS-T for one hour. Blots were then probed against primary antibodies overnight at 4°C. The following day, blots were washed with TBS-T and then HRP-conjugated secondaries were applied against the respective primary for one hour at room temperature. All blots were then developed with enhanced chemiluminescence (EMD Millipore, WBKLS0100) for imaging on a ChemiDoc Imager (BioRad).

**Behavior**

**Behavioral Milestones**

The Hill, et al. 2008 paradigm was used to measure the development of milestones across the first 2 weeks of life following mTBI. All behavior equipment was cleaned with 70% ethanol between mice. The following behaviors measured are detailed below:

- Surface righting (SR) was used to assess labyrinthine and body righting mechanisms, strength, and coordination. The pup was placed on its back and had to flip over onto all four paws in less than 1 second.
- Negative geotaxis (NG) was used to assess labyrinthine reflex and body righting mechanisms, strength, and coordination. The pup was placed facing downwards on a gridded screen at a 45-degree angle. The pup must turn 180 degrees in less than 30 seconds.
- Cliff aversion (CA) was used to assess labyrinthine reflex, strength, and coordination. The pup was placed on top of a pipette box, with its front paw and snout over the edge. The pup must back away from the edge in less than 30 seconds.
- Rooting (R) was used to assess tactile reflex and motor coordination. A small filament was twisted and used to brush against the side of the pup’s head three times. The pup must turn its head in the direction of the filament to be deemed a pass.
- The forelimb grasp (FG) was used to assess strength. The pup must hold itself up with its forepaws on a suspended bar for at least 1 second.
- The auditory startle (AS) response was used to assess auditory reflex. The tester performed a handclap approximately 10 cm from the pup’s head. The pup must respond with an instant, involuntary jump to be considered a pass.
- The ear twitch (ET) was used to assess tactile reflex. Again, a small filament was twisted and used to brush against the tip of the pup’s ear. If the pup flattened its ear against its head, it was deemed a pass.
- The open field traversal test (OF) was used to assess locomotion and extinguishing of pivoting behavior. The pup was placed on a plastic sheet with a 13 cm diameter outlined circle. The pup must travel out of the circle, with all four paws, in less than 30 seconds.
- Pups were checked daily for two fully opened eyes (EO).
- Finally, air righting (AR) was used to assess labyrinthine and body righting mechanisms and coordination. The pup was held upside down and suspended 10.5 cm over the cage by the experimenter. The pup was then released and must land right side up on all four paws to pass.

**Ultrasonic Vocalization Analysis**

USV call files were exported as .wav files and then analyzed using the MATLAB program, DeepSqueak (Version 3.1.0). Within DeepSqueak, neural network “Mouse Detector YOLO R2.mat” was selected and then audio .wav files were imported to “load calls” to produce a .mat script. Individual .mat files were then run using “detect calls”. USV calls were then either accepted or rejected as background noise by a blinded experimenter and data was then exported into an Excel file. USV data were analyzed for the total number of calls elicited, as well as the latency (seconds) until the first isolation-induced call for each mouse.

Call typologies from each .wav file were also classified using DeepSqueak (Version 3.1.0). Briefly, contour clustering of USV call classifications was performed by DeepSqueak software by selecting the following prompts: “Tools”, “Call Classification”, “Unsupervised Clustering”, “Contour Parameters”, “No”, open .mat file, ranking clustering weight in order: shape, frequency, duration, “Elbow Optimized”, Max Clusters “100”, Replicates “3”. A blinded experimenter refined the call classifications as described by Scattoni et al., 2008 into eleven clusters named: complex, harmonics, two-syllable, upward, downward, chevron, short, composite, frequency steps, flat, and background noise.

**Supplemental Results**

**White matter loss extends to the contralateral hemisphere**

In the contralateral hemisphere, MBP loss was not found at: 4.35 mm (F (3, 17) = 1.243, p= 0.325) (**Supp Fig. 1A**) or 4.47 mm (F (3, 22) = 2.654, p= 0.074) (**Supp Fig. 1B**). There were differences at 4.59 mm (F (3, 26) = 10.43, p< 0.001) (**Supp Fig. 1C**) and 4.71mm (F (3, 14) = 3.488, p= 0.045) (**Supp Fig. 1D**). Post-hoc comparisons revealed at level 4.59 mm that .02J mTBI caused a loss of MBP compared to shams and .01J mice (p= 0.012 and p< 0.001, respectively). There were no differences in the ipsilateral: contralateral ratio of MBP staining at any anatomical level: 4.35 mm (F (3, 18) = 2.015, p= 0.148) (**Supp Fig. 1E**), 4.47 mm (F (3, 22) = 1.045, p=0.393) (**Supp Fig. 1F**), 4.59 mm (F (3, 29) = 0.366, p= 0.778) (**Supp Fig. 1G**), and 4.71 mm (F (3, 14) = 1.012, p= 0.379) (**Supp Fig. 1H**).

**IBA1+ percent stain across hemispheres**

Overall, we observed no overt signs of microgliosis, or increased IBA1 staining, in the ipsilateral or contralateral hemisphere, respectively, at any level: 4.35 mm: (F (3, 27) = 1.099, p= 0.367) and (F (3, 26) = 0.634, p= 0.600) (**Supp Fig. 2A**); 4.47 mm (F (3, 28) = 2.170, p= 0.114) and (F (3, 29) = 1.616, p= 0.207) (**Supp Fig. 2B**); and at 4.59 mm: (F (3, 36) = 1.019, p= 0.396) and (F (3, 36) = 0.796, p= 0.504) (**Supp Fig. 2C**).

**Lack of mTBI-induced astrogliosis at 5 dpi**

To determine if subacute astrogliosis occurs after mTBI we stained for GFAP+ astrocytes (**Supp** **Fig. 3A**) to determine if there was higher DAB+ area coverage (concentrated GFAP+ cells) in the ipsilateral compared to contralateral parietal cortex. Overall, we saw no significant differences in GFAP+ percent area in the ipsilateral: contralateral ratio at any level: 4.35 mm (F (3, 15) = 0.828, p= 0.500), 4.47 mm (H= 3.868, p= 0.281), and 4.59 mm (F (3, 13) = 2.861, p= 0.078) (**Supp** **Fig. 3B-D**), nor in the percent area of GFAP staining in ipsilateral or contralateral hemispheres: 4.35 mm: (H= 3.313, p= 0.346) and (H= 6.856, p= 0.077); 4.47 mm (H= 3.00, p= 0.046) and (H= 2.897, p= 0.474); and at 4.59 mm (H= 2.069, p= 0.632) and (H= 4.539, p= 0.193) (**Supp. Fig 3E-G**).

**Magnitude of behavioral milestone delays across injury severity**

Male .02J mTBI mice reached 100% pass one day after shams in six tasks. Male .04J mTBI mice were delayed by one day in three tasks, delayed by two days in three tasks, and delayed by three days in one task. (**Supp. Table 2**). Female .02J mTBI mice were delayed by one day in three tasks and delayed by three days in one task. Female .04J mTBI mice were delayed by one day in four tasks (**Supp. Table 2**).
