# Supplementary material for: Early-Life Mild Traumatic Brain Injury Alters Neurodevelopment and Behavior in Mice
Source: Neurotrauma Rep. 2025 Jun 30;6(1):465–79. doi: 10.1089/neur.2025.0016 (PMC12270539; doi:10.1089/neur.2025.0016)
Supplement: Supplementary Figure S1 [file neur.2025.0016_supplementaryfigures1.docx]

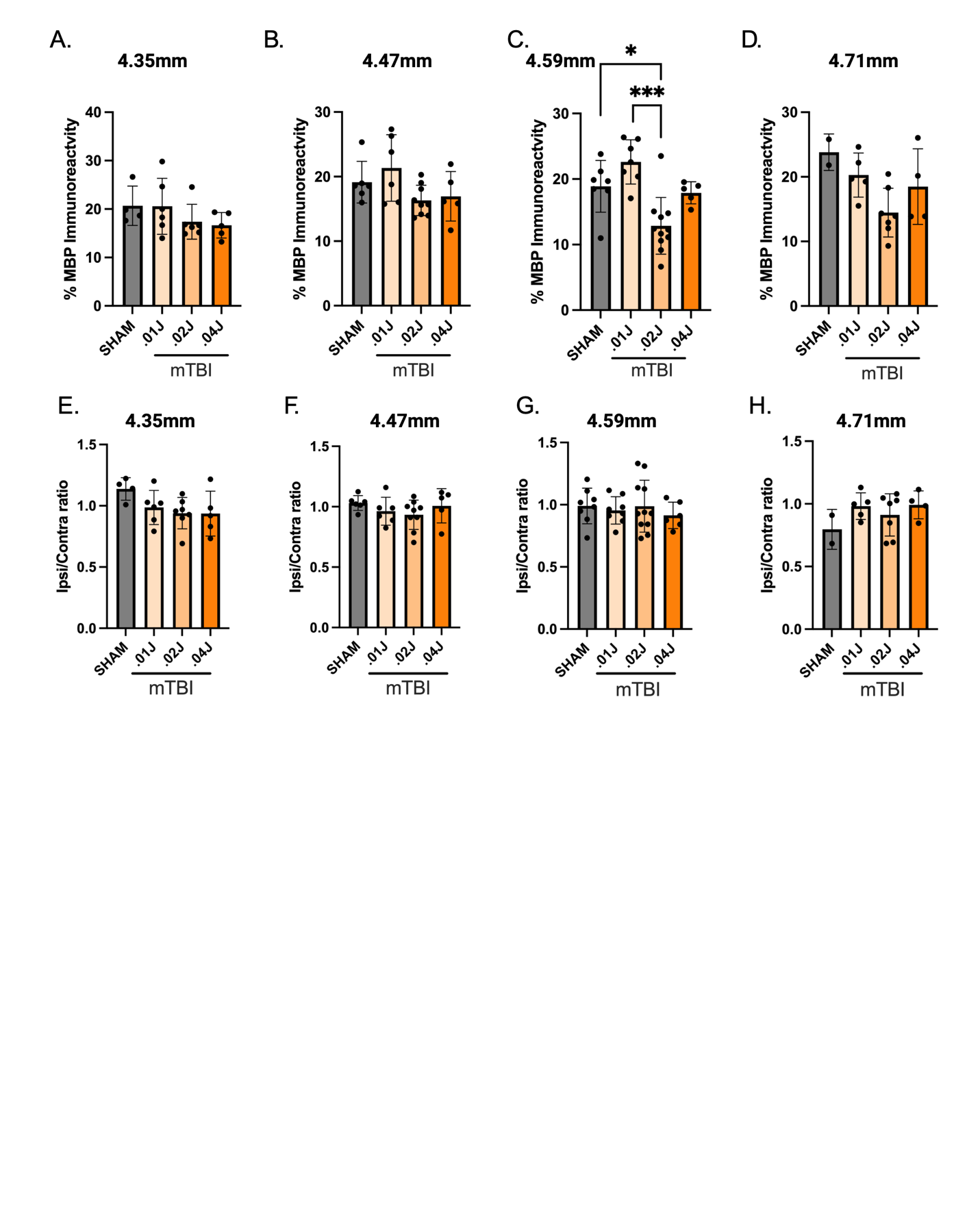


**Supplemental Figure 1**

**MBP Immunoreactivity in the Contralateral Hemisphere:** **A**. Percent area of MBP immunoreactivity in the contralateral hemisphere at 4.35 mm (N, sham: 4, .01J mTBI: 6, .02J mTBI: 6, .04J: mTBI: 5), **B**. at 4.47 mm (N, sham: 6, .01J mTBI: 6, .02J mTBI: 9, .04J: mTBI: 5), **C**. at 4.59 mm (N, sham: 7, .01J mTBI: 7, .02J mTBI: 11, .04J mTBI: 5), and **D**. at 4.71 mm (N, sham: 2, .01J mTBI: 5, .02J mTBI: 7, .04J mTBI: 4). One-way ANOVAs with Tukey’s multiple comparisons test: * p< 0.05, *** p< 0.001. **E**. Ratio of ipsilateral to contralateral percent area of MBP immunoreactivity at 4.35 mm (N, sham: 4, .01J mTBI: 6, .02J mTBI: 7, .04J mTBI: 5), **F**. at 4.47 mm (N, sham: 6, .01J mTBI: 6, .02J mTBI: 9, .04J mTBI: 5), **G**. at 4.59 mm (N, sham: 7, .01J mTBI: 7, .02J mTBI: 11, .04J mTBI 5), and **H**. at 4.71 mm (N, sham: 2, .01J mTBI: 5, .02J mTBI: 7, .04J mTBI: 4). One-way ANOVAs with Tukey’s multiple comparisons test. Data presented as mean $\pm$ SD.
