# Supplementary material for: Early-Life Mild Traumatic Brain Injury Alters Neurodevelopment and Behavior in Mice
Source: Neurotrauma Rep. 2025 Jun 30;6(1):465–79. doi: 10.1089/neur.2025.0016 (PMC12270539; doi:10.1089/neur.2025.0016)
Supplement: Supplementary Figure S2 [file neur.2025.0016_supplementaryfigures2.docx]

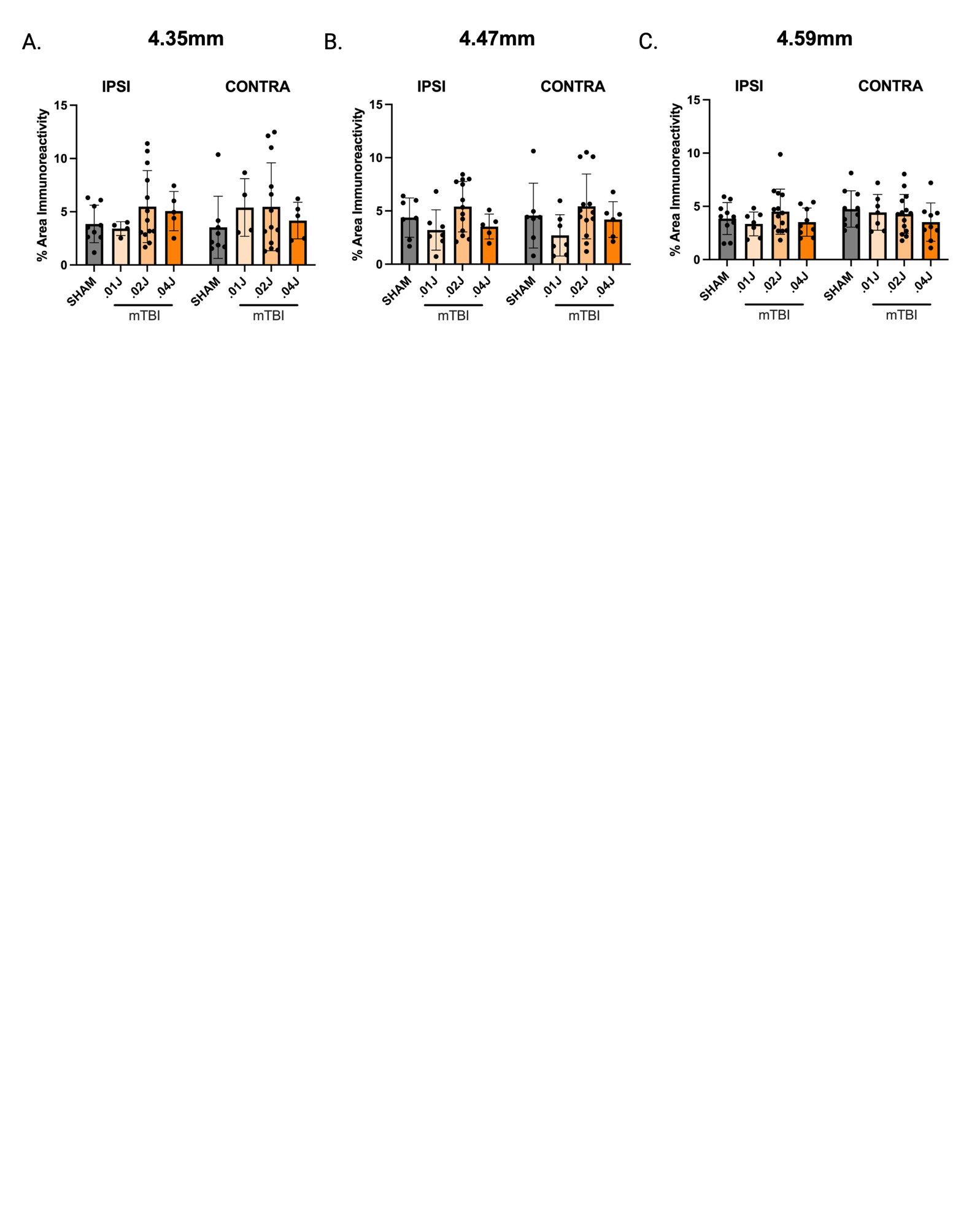


**Supplemental Figure 2**

**IBA-1 Immunoreactivity:** **A**. Percent area of IBA-1 immunoreactivity in the ipsilateral and contralateral hemispheres at 4.35 mm (N, sham: 9, .01J mTBI: 4, .02J mTBI: 13, .04J: mTBI: 5), **B**. at 4.47 mm (N, sham: 7, .01J mTBI: 7, .02J mTBI: 13, .04J mTBI: 6), and **C**. at 4.59 mm (N, sham: 10, .01J mTBI: 7, .02J mTBI: 14, .04J mTBI: 9). One-way ANOVAs with Tukey’s multiple comparisons test. Data presented as mean $\pm$ SD.
