# Supplementary material for: Early-Life Mild Traumatic Brain Injury Alters Neurodevelopment and Behavior in Mice
Source: Neurotrauma Rep. 2025 Jun 30;6(1):465–79. doi: 10.1089/neur.2025.0016 (PMC12270539; doi:10.1089/neur.2025.0016)
Supplement: Supplementary Figure S3 [file neur.2025.0016_supplementaryfigures3.docx]

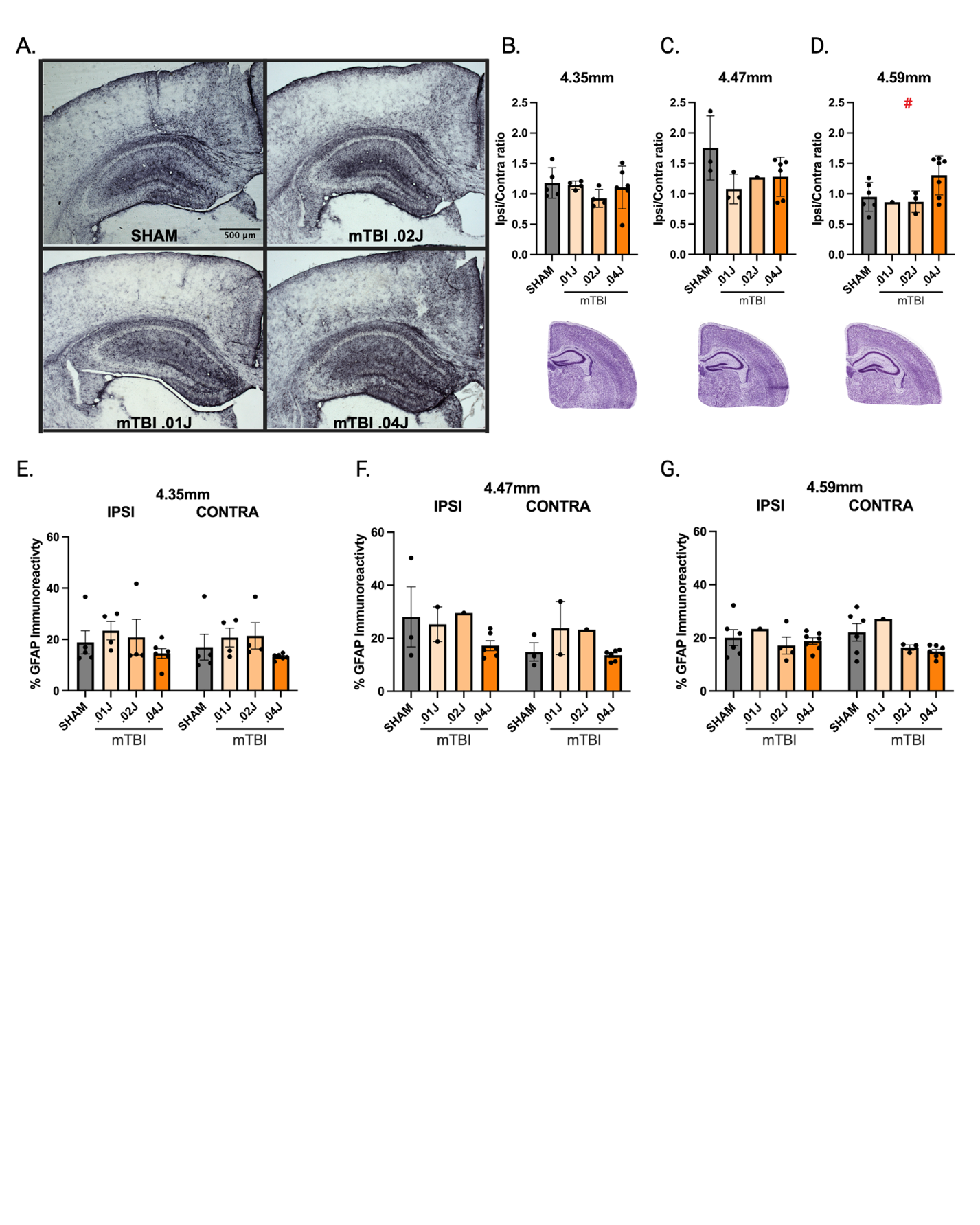


**Supplemental Figure 3**

**GFAP Immunoreactivity at 5 dpi:** **A.** Representative images (5x) of GFAP immunoreactivity at anatomical level 4.59 mm. **B**. Ratio of ipsilateral to contralateral percent area of GFAP immunoreactivity at 4.35 mm (N, sham: 5, .01J mTBI: 4, .02J mTBI: 4, .04J: mTBI: 6), **C**. at 4.47 mm (N, sham: 3, .01J mTBI: 3, .02J mTBI: 1, .04J: mTBI: 6), and **D**. at 4.59 mm (N, sham: 6, .01J mTBI: 1, .02J mTBI: 3, .04J: mTBI: 7). One-way ANOVAs with Tukey’s multiple comparisons test: # p< 0.1. **E**. Percent area GFAP immunoreactivity in the ipsilateral and contralateral hemispheres at 4.35 mm (N, sham: 5, .01J mTBI: 4, .02J mTBI: 4, .04J: mTBI: 6), **F**. at 4.47 mm (N, sham: 3, .01J mTBI: 2, .02J mTBI: 1, .04J mTBI: 6), and **G**. at 4.59mm (N, sham: 6, .01J mTBI: 1, .02J mTBI: 4, .04J: mTBI: 7). Kruskal-Wallace test with Dunn’s post-hoc comparisons. Data presented as mean $\pm$ SD.
