# Supplementary material for: Early-Life Mild Traumatic Brain Injury Alters Neurodevelopment and Behavior in Mice
Source: Neurotrauma Rep. 2025 Jun 30;6(1):465–79. doi: 10.1089/neur.2025.0016 (PMC12270539; doi:10.1089/neur.2025.0016)
Supplement: Supplementary Tables [file neur.2025.0016_supplementarytables.docx]

SUPPLEMENTAL TABLE 1

Primary antibodies used in study.

|  | Primary IgG | Concentration | Company | CAT # |
| --- | --- | --- | --- | --- |
| IHC | Myelin Basic Protein (MBP) | 1: 500 | Aves Labs | MBP |
|  | Ionized calcium binding adaptor molecule 1 (IBA-1) | 1: 1,000 | Wako Fujifilm | 019-19741 |
|  | Glial Fibrillary Acidic Protein (GFAP) | 1: 1,000 | Cell Signaling | #80788 |
| WB | CX3C motif chemokine receptor 1 (CX3CR1) | 1ug/ml | R&D Systems | AF5825 |
|  | Complement component 1qa (C1q) | 1: 1,000 | Abcam | ab155052 |
|  | Complement component 3 (C3) | 1: 1,000 | Abcam | ab200999 |
|  | $\beta$- actin | 1: 20,000 | Invitrogen | MA1-91399 |

SUPPLMENTAL TABLE 2

| Developmental  Milestone | | MALE  SHAM mTBI .02J mTBI .04J | | | FEMALE  SHAM mTBI .02J mTBI .04J | | |
| --- | --- | --- | --- | --- | --- | --- | --- |
| Negative Geotaxis | 10  (9.1 ± 0.3) | | 11  (9.2 ± 0.6) | 12  (9.7 ± 1.3) | 12  (9.7 ± 1.2) | 11  (11.6 ± 0.6) | 12  (12.5 ± 1.1) |
| Auditory Startle | 11  (10.5 ± 0.5) | | 12  (10.5 ± 0.9) | 13  (11 ± 1.1) | 13  (11 ± 1.4) | 13  (10.2 ± 1.4) | 13  (9.9 ± 0.9) |
| Cliff Aversion | 11  (9.2 ± 0.6) | | 12  (9.5 ± 0.9) | 12  (9.4 ± 1.0) | 11  (9.3 ± 0.8) | 12  (9.6 ± 1.1) | 11  (9.2 ± 0.6) |
| Forelimb Grasp | 11  (9.9 ± 0.7) | | 12  (10.6 ± 0.9) | 14  (10.3 ± 1.5) | 12  (10.8 ±1.0) | 13  (10.6 ± 1.1) | 13  (10.1 ± 1.4) |
| Rooting | 12  (9.8 ± 1.0) | | 12  (10.2 ± 1.2) | 14  (10.6 ± 1.6) | 12  (10 ± 1.3) | 12  (9.9 ± 1.0) | 12  (9.9 ± 0.7) |
| Open Field | 13  (11.5 ± 1.1) | | 13  (11.6 ± 0.9) | 14  (12.2 ± 1.2) | 12  (11.2 ± 0.8) | 15  (12.4 ± 0.6) | 13  (12 ± 0.6) |
| Ear Twitch | 13  (12 ± 0.4) | | 14  (12.2 ± 0.8) | 12  (11.3 ± 0.8) | 14  (12.3 ± 0.8) | 14  (11.9 ± 0.7) | 14  (12.1 ± 0.7) |
| Surface Righting | 13  (11.8 ± 1.3) | | 14  (12.4 ± 1.6) | 14  (11.4 ± 1.6) | 13  (11.7 ± 1.5) | N/A | 14  (12.5 ± 0.8) |
| Air Righting | 15  (13.3 ± 1.2) | | 14  (12.6 ± 0.8) | N/A | 14  (13 ± 1.0) | 15  (12.8 ± 1.2) | 15  (13.5 ± 1.2) |
| Eye Opening | 16  (14.8 ± 0.6) | | 16  (14.8 ± 0.6) | 16  (14.8 ± 0.6) | 16  (14.8 ± 0.8) | 16  (14.8 ± 0.6) | 16  (14.9 ± 0.6) |

Postnatal day of 100% pass for each group. Parentheses indicate group average ± SD postnatal day of pass. N/A= 100% of mice never successfully passed task by study endpoint.

SUPPLEMENTAL TABLE 3- AVERAGE PERCENTAGE OF TYPOLOGY CALLS MADE IN MALES

| Typologies | MEAN ±SD  SHAM mTBI .02J mTBI .04J | | | Statistic | P value |
| --- | --- | --- | --- | --- | --- |
| “one-syllable” | | | | | |
| downward | 25.76 ± 11.39 | 24.81 ± 11.65 | 21.77 ± 8.10 | H= 0.366 | 0.833 |
| short | 21.65 ± 18.66 | 15.73 ± 9.58 | 13.66 ± 8.84 | H= 0.822 | 0.663 |
| chevron | 18.79 ± 11.90 | 18.16 ± 4.16 | 14.51 ± 8.26 | H= 3.284 | 0.194 |
| upward | 15.43 ± 11.47 | 15.30 ± 8.84 | 12.69 ± 8.49 | H= 0.373 | 0.830 |
| complex | 3.66 ± 2.90 | 7.96 ± 4.18 * | 5.44 ± 4.51 | H= 7.215 | 0.027 |
| flat | 0.00 ± 0.00 | 0.05 ± 0.18 | 0.31 ± 0.94 | H= 1.367 | 0.505 |
| “multi-syllable” | | | | | |
| two syllable | 5.81 ± 5.38 | 7.80 ± 3.39 | 8.49 ± 3.38 | H= 3.228 | 0.199 |
| frequency steps | 3.58 ± 4.06 | 4.88 ± 3.66 | 4.01 ± 2.84 | H= 1.248 | 0.536 |
| composite | 2.67 ± 2.62 | 2.57 ± 1.96 | 6.08 ± 4.44 | H= 4.445 | 0.108 |
| harmonics | 2.65 ± 2.80 | 2.73 ± 2.44 | 12.58 ± 15.05 | H= 4.463 | 0.1074 |

Dunn’s multiple comparisons test: * = p< 0.01 compared to sham.

SUPPLEMENTAL TABLE 4- AVERAGE PERCENTAGE OF TYPOLOGY CALLS MADE IN FEMALES

| Typologies | MEAN ±SD  SHAM mTBI .02J mTBI .04J | | | Statistic | P value |
| --- | --- | --- | --- | --- | --- |
| “one-syllable” | | | | | |
| downward | 20.87 ± 12.92 | 16.27 ± 10.62 | 30.60 ± 15.21 | H= 5.871 | 0.053 |
| chevron | 14.96 ± 13.07 | 13.79 ± 7.50 | 17.70 ± 10.62 | H= 1.167 | 0.558 |
| short | 14.50 ± 7.28 | 18.48 ± 14.63 | 13.79 ± 6.74 | H= 0.051 | 0.975 |
| upward | 14.34 ± 8.81 | 14.60 ± 9.46 | 10.66 ± 6.31 | H= 1.130 | 0.568 |
| complex | 6.00 ± 2.41 | 8.72 ± 5.25 | 4.68 ± 4.28 | H= 4.970 | 0.083 |
| flat | 0.18 ± 0.34 | 0.69 ± 1.89 | 0.03 ± 0.10 | H=1.24 | 0.536 |
| “multi- syllable” | | | | | |
| two syllable | 8.37 ± 4.07 | 9.93 ± 8.04 | 6.82 ± 4.13 | H= 3.579 | 0.167 |
| composite | 7.53 ± 9.15 | 3.52 ± 4.80 | 3.88 ± 5.31 | H= 2.47 | 0.292 |
| frequency steps | 6.68 ± 3.85 | 7.02 ± 7.20 | 5.54 ± 5.75 | H= 0.846 | 0.655 |
| harmonics | 6.57 ± 4.99 | 6.99 ± 9.20 | 7.89 ± 10.06 | H= 0.459 | 0.795 |
